# Oxidized Albumin Induces Renal Tubular Cell Death and Promotes the Progression of Renal Diseases Through Ferroptosis

**DOI:** 10.3390/ijms26135924

**Published:** 2025-06-20

**Authors:** Yingyu Zhang, Rui Jiang, Zhuheng Shi, Yang Sui, Jie Cheng, Mika Suda, Manabu Niimi, Kun Gao, Jianglin Fan, Jian Yao

**Affiliations:** 1 Division of Molecular Signaling, Department of the Advanced Biomedical Research, Interdisciplinary Graduate School of Medicine, University of Yamanashi, Yamanashi 409-3898, Japan; g22dima2@yamanashi.ac.jp (Y.Z.); doctorrui1992@163.com (R.J.); g23dima1@yamanashi.ac.jp (Z.S.); sunnysuiyang@163.com (Y.S.); g23dima2@yamanashi.ac.jp (J.C.); sdmika@yamanashi.ac.jp (M.S.); kungao@njucm.edu.cn (K.G.); 2 Division of Molecular Pathology, Interdisciplinary Graduate School of Medicine, University of Yamanashi, Yamanashi 409-3898, Japan; manabun@yamanashi.ac.jp

**Keywords:** oxidative stress, albumin, oxidized albumin, ferroptosis, kidney injury

## Abstract

Oxidative stress plays a crucial role in disease pathogenesis. While reactive oxygen species (ROS) directly cause cellular injury, emerging evidence suggests oxidatively modified proteins like albumin may also contribute significantly to tissue damage. Although oxidized albumin (ox-Alb) is linked to renal pathology, the direct effects and mechanisms of ox-Alb on renal cell injury remain unclear. This study was created to address these questions. In mouse models of renal injury initiated by vitamin C/copper or ischemia/reperfusion, levels of serum ox-Alb were significantly elevated. The treatment of albumin with copper/vitamin C increased Alb carbonylation and reduced the number of sulfhydryl groups, causing Alb oxidation. In cultured renal tubular epithelial NRK-52E cells, ox-Alb triggered cell death, associated with increased intracellular albumin accumulation—enhanced cellular protein carbonylation, and p38 MAPK activation. Notably, ox-Alb induced ferroptosis, evidenced by decreased GPX4 and xCT, increased ACSL4, elevated iron and lipid peroxidation, and suppression by deferoxamine and liproxstatin-1. In vivo, administration of ox-Alb exacerbated doxorubicin-induced nephropathy, as indicated by the elevated BUN, creatinine, and proteinuria, and intensified renal ferroptotic responses, including altered GPX4 and ACSL4. Our findings demonstrate that ox-Alb induces renal cell ferroptosis and promotes renal disease progression, suggesting its pivotal pathogenic role in oxidative stress-related kidney diseases.

## 1. Introduction

Acute kidney injury (AKI) and chronic kidney disease (CKD) are significant global health challenges that affect millions of patients worldwide, resulting in substantial morbidity and mortality. These conditions are caused by a complex array of pathological processes including ischemia/reperfusion injury, exposure to nephrotoxicants, inflammatory responses, and diabetes [1]. Among the various mechanisms implicated in the pathogenesis of AKI and CKD, oxidative stress has emerged as a pivotal mediator of renal injury [2,3].

Oxidative stress is characterized by an imbalance between the production of reactive oxygen species (ROS) and the capacity of cellular antioxidant defense systems to neutralize these ROS and repair the resulting damage. In the kidney, ROS are generated through several key molecular mechanisms, including the dysfunction of the mitochondrial electron transport chain, the activation of NADPH oxidases, the activity of xanthine oxidase, and uncoupled nitric oxide synthases. Additionally, the kidneys are subjected to circulating free radicals, oxidative metabolites, and oxidatively modified biomolecules, making them particularly susceptible to oxidative damage due to their high metabolic demands and critical role in blood filtration and urine formation [2,3,4].

Recent evidence indicates that the pathogenic effects of oxidative stress extend well beyond direct molecular damage. Oxidatively modified biomolecules are not merely bystanders; they actively participate in cellular injury processes and serve as drivers of disease pathogenesis [5,6,7,8,9]. Notably, oxidatively modified proteins have been recognized for their potential role in the progression of both acute and chronic kidney diseases. Proteins are especially vulnerable to oxidative modifications because amino acid side chains are susceptible to oxidation. These non-enzymatic alterations, including carbonylation, dityrosine crosslinking, disulfide bond formation, sulfhydryl oxidation, nitration, chlorination, etc., disrupt protein structures and impair biological functions, leading to the accumulation of dysfunctional proteins within cells [10,11]. While the impact of intracellular oxidized proteins is well-documented in various oxidative diseases, the effects of extracellular oxidized proteins on cell injury and disease progression are less understood.

Albumin (Alb), due to its abundance in the bloodstream and unique structural features, is particularly prone to oxidative modifications [5,8]. The single free cysteine thiol at residue 34 (Cys34) acts as the primary extracellular redox buffer, accounting for approximately 80% of all free thiol groups within the plasma. Under normal conditions, Cys34 enables Alb to perform essential antioxidant functions. However, under oxidative stress, the equilibrium shifts towards the formation of oxidized Alb (ox-Alb). Consequently, elevated levels of ox-Alb, featuring oxidized Cys34 and other modifications, have been detected in various disease states associated with oxidative stress [5,7,8,12]. Beyond its role as a biomarker, the specific pathological effects of ox-Alb are not fully understood.

The kidneys, particularly the proximal tubular epithelial cells, are highly susceptible to protein overload due to their role in reabsorbing proteins filtered by the glomerulus [4,8,13,14]. It has been documented that protein overload is an important pathogenic factor contributing to the initiation and progression of kidney diseases. However, most studies have primarily focused on native proteins. The impacts and mechanisms of oxidized proteins such as Alb on kidney disease pathogenesis remain to be elucidated.

This study aims to answer whether ox-Alb could contribute to renal cell injury and the progression of kidney disease. Specifically, the study will (1) demonstrate the presence of oxidized serum proteins, particularly Alb, in oxidative models of kidney injury; (2) investigate the cytotoxic effects of ox-Alb on cultured renal tubular cells and its role in the progression of kidney disease in a doxorubicin (DOX)-induced kidney injury model; and (3) explore the molecular mechanisms underlying ox-Alb-induced cell injury, with a particular focus on oxidative stress and ferroptosis—a form of iron-dependent, lipid peroxidation-driven cell death that has been shown to be implicated in various diseases, including kidney injury [15,16,17]. The findings from this study will provide novel insights into the role of oxidized proteins in renal cell damage and disease progression and propose potential strategies for preventing or treating kidney diseases associated with oxidative stress.

## 2. Results

### 2.1. Elevated Serum Alb Oxidation in Models of Oxidative Renal Injury

**Figure 1:**
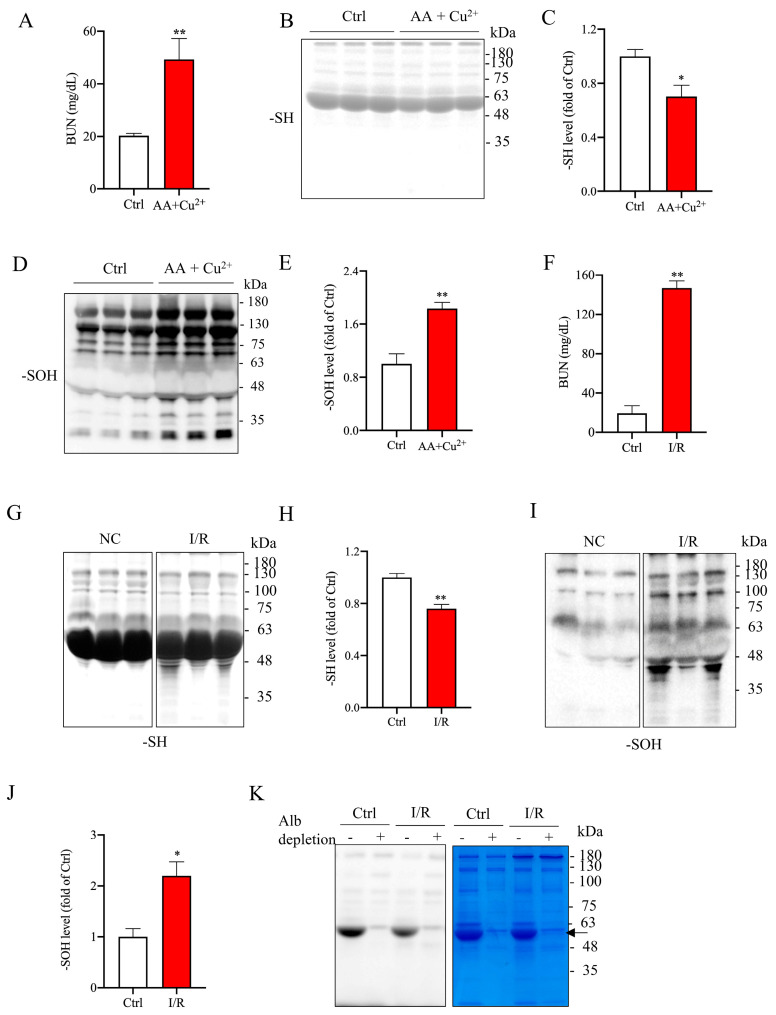
Association of oxidative kidney injury with elevated serum protein oxidation. (**A**) Serum BUN levels in mice following administration of AA (100 mg/kg) and Cu^2+^ (1 mg/kg) for 6 days. Data are expressed as mg/dl and presented as mean ± standard error of the mean (SEM) (*n* = 3). Statistical differences were performed using Student’s *t*-test; ** *p* < 0.01 vs. control. (**B**–**E**) Western blot analysis of -SH levels and -SOH formation in serum proteins. Quantitative analysis of bands from panels (**B**) and (**D**) is shown in panels (**C**) and (**E**), respectively. Values are expressed as fold change relative to the control (mean ± SEM; n = 3; * *p* < 0.05 vs. control; ** *p* < 0.01 vs. control as analyzed with Student’s *t*-test). (**F**) Serum BUN levels in mice subjected to renal ischemia/reperfusion measured at the 24-h point (n = 3; * *p* < 0.05 vs. control; ** *p* < 0.01 vs. control). (**G**,**I**) Representative Western blots depicting the levels of -SH and -SOH groups in serum proteins. (**H**,**J**) Densitometric analysis of the band intensity at the MW of 48~63 in (**G**) and (**I**), respectively. Data are presented as fold change relative to sham control (n = 3/group; * *p* < 0.05 vs. control; ** *p* < 0.01 vs. control). (**K**) Loss of the major -SH signal in serum proteins following albumin depletion. Serums from normal and renal ischemia/reperfusion mice were collected. Serum albumin was depleted using an albumin depletion reagent. The -SH level in serum proteins was analyzed with a maleimide-labelling assay. EZ Blue staining was performed to confirm the disappearance of the albumin band (arrow). Note the loss of the major protein -SH bands after depletion of serum albumin (arrow).

Increased serum Alb oxidation has been reported in various models of renal diseases [5,7,8,18]. In our studies on oxidative renal injury induced by L-ascorbic acid (AA) plus copper (II) sulfate (Cu^2+^) or renal ischemia/reperfusion (I/R), we also observed a strong correlation between renal dysfunction and elevated serum protein oxidation [19,20]. Consistent with previous findings, we confirmed the presence of serum protein oxidation in our current study. As shown in Figure 1A–E, administration of Cu^2+^ plus AA to mice for 6 days led to renal dysfunction, as evidenced by elevated blood urea nitrogen (BUN). This renal impairment was accompanied by reduced protein sulfhydryl (-SH) levels and increased protein sulfenic acid (-SOH) formation at the major band around 60 kDa. A similar change was also observed in the renal injury model induced by I/R (Figure 1F–J). These observations thus confirm the existence of serum protein oxidation in the renal injury models.

Given the molecular weight, abundance in serum, richness in -SH groups, and high susceptibility to oxidative modification [5,8], we speculated that Alb was the primary protein undergoing oxidation. To test this hypothesis, we used an Alb depletion kit to remove Alb from serum samples and subsequently examined the changes in protein -SH levels. As shown in Figure 1K, Alb depletion resulted in the disappearance of the major protein band in the EZ Blue staining, which was accompanied by a loss of the predominant -SH band in the maleimide-labeling assay. Notably, the significant difference in protein -SH levels observed between normal and I/R serums disappeared (Figure 1K). This observation thus confirms that Alb is the primary serum protein to be oxidized during oxidative kidney injury.

### 2.2. Preparation and Characterization of Oxidized Albumin

**Figure 2:**
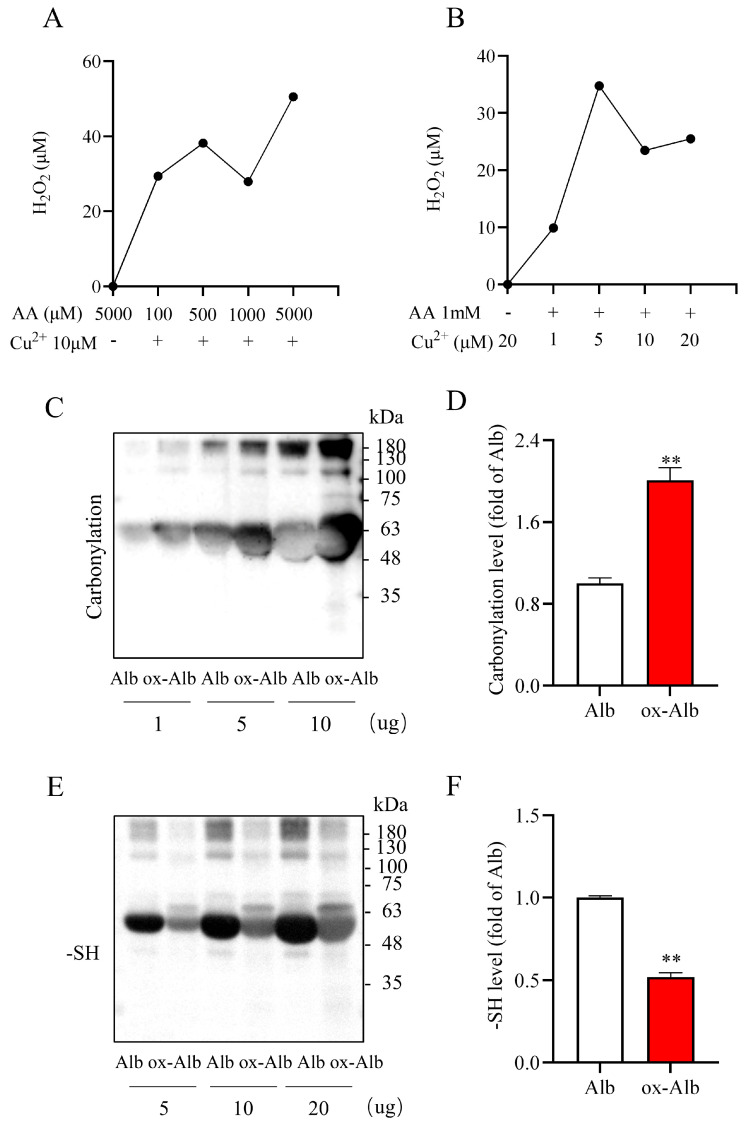
Preparation and characterization of in vitro ox-Alb. (**A**,**B**) H_2_O_2_ production from the reaction of the indicated concentrations of AA and Cu^2+^ after 30 min of co-incubation was measured using a commercial kit as described in the Materials and Methods section. The data shown are the average H_2_O_2_ concentration of two duplicate wells. (**C**–**F**) Treatment of Alb with 0.4 M AA and 2 mM Cu^2+^ at 4 °C for 48 h. A portion of Alb at the indicated concentrations was assayed for the levels of protein carbonylation and -SH group. Note the increased carbonylation and decreased -SH in oxidized samples. Quantitative analysis of the changes in (**C**) and (**E**) is presented in (**D**) and (**F**), respectively. Data are expressed as fold changes relative to Alb control (mean ± SEM; n = 3; ** *p* < 0.01 as analyzed with Student’s *t*-test).

To determine the potential role and mechanism of ox-Alb in renal disease pathogenesis, we generated ox-Alb by exposing Alb to AA plus Cu^2+^ solution. In our previous study [20], we demonstrated that AA and Cu^2+^ in combination initiated a Fenton reaction, generating a substantial amount of hydrogen peroxide (H_2_O_2_). Here, we reconfirmed the findings (Figure 2A,B): AA plus Cu^2+^ caused a concentration-dependent generation of H_2_O_2_.

Using this property of AA plus Cu^2+^, we oxidized Alb and confirmed its oxidation. For this purpose, we detected the carbonyl formation and -SH groups in Alb due to their simplicity in detection and the established roles as oxidative markers [10,21]. As shown in Figure 2C–F, treatment of Alb with AA plus Cu^2+^ significantly increased Alb carbonylation and decreased its-SH level, confirming the successful induction of Alb oxidation.

### 2.3. Cytotoxic Effects of Ox-Alb on Renal and Intestinal Epithelial Cells

**Figure 3:**
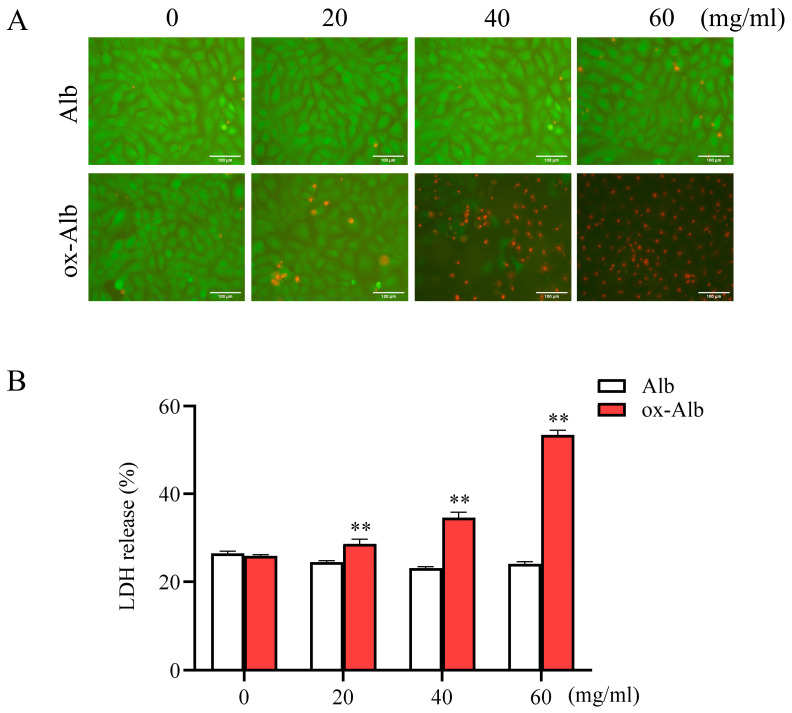
Impact of ox-Alb on renal tubular cell viability. Renal tubular epithelial NRK-E52 cells were exposed to the indicated concentrations of native or ox-Alb for 24 h. Cell viability was assessed via Calcein-AM/PI staining (**A**) and LDH release assay (**B**). Panel (**B**) data represent percentage changes relative to total LDH release (mean ± SEM; n = 4; ** *p* < 0.01 as analyzed with one-way ANOVA). Scale bar = 100 mm.

With the prepared ox-Alb, we examined the potential cytotoxic effects on cultured renal tubular epithelial NRK-52E cells. Figure 3 shows that native Alb did not affect cell viability at concentrations up to 60 mg/mL, as indicated by calcein acetoxymethyl (AM)/propidium iodide (PI) staining and lactate dehydrogenase (LDH) release. In contrast, ox-Alb significantly increased cell death. It induced a substantial reduction in the number of Calcein-AM-positive living cells and an apparent increase in the number of PI-positive dead cells. Consistently, it significantly elevated LDH release (Figure 3). These results indicate that ox-Alb is markedly more cytotoxic to renal tubular cells.

Other than renal tubular epithelial NRK-E52 cells, we also tested the cytotoxicity of ox-Alb on human intestinal epithelial Caco-2 cells and found that it similarly caused a significantly higher level of cell death in Caco-2 cells than normal Alb (Supplementary Figure S1). It appears that the cytotoxicity of ox-Alb is not cell type-specific.

### 2.4. Ox-Alb Induces Alb Accumulation and Oxidative Stress in Cultured Renal Cells

**Figure 4:**
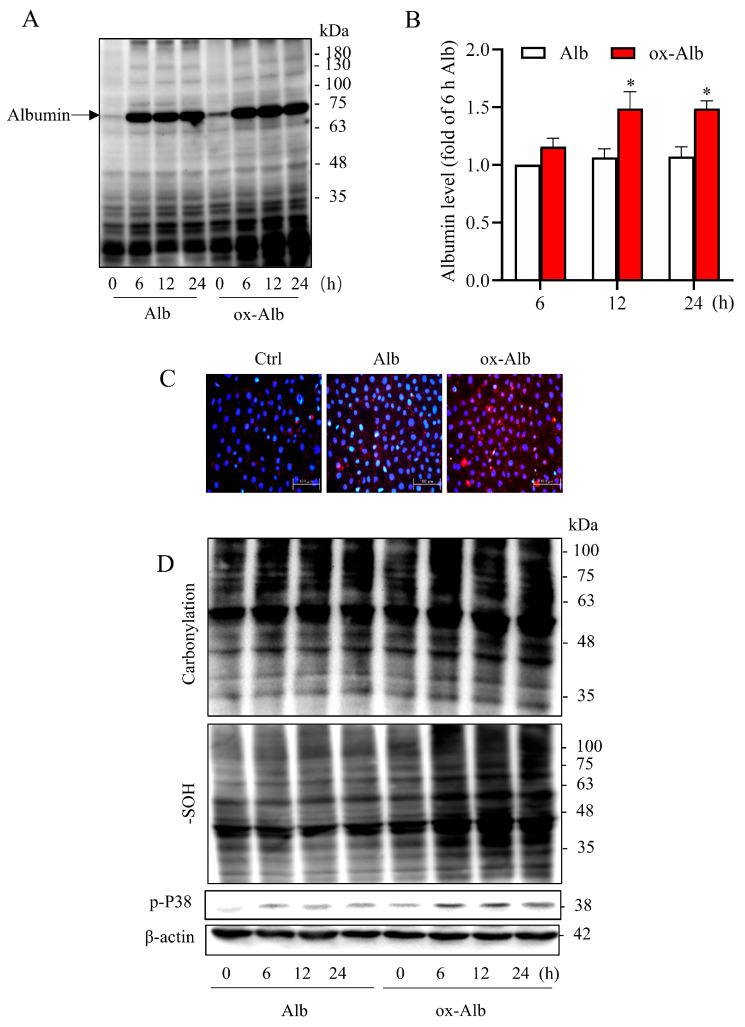
Cellular Alb level and oxidative status following incubation with Alb. (**A**–**C**) Analysis of Alb uptake in NRK cells exposed to 20 mg/mL of control or ox-Alb for specified times (**A**,**B**) or 24 h (**C**). Alb levels in cellular lysates were determined by Western blot (**A**,**B**) and immunofluorescence staining (**C**). Densitometric quantification from (**A**) is shown in (**B**) as fold change relative to the level of Alb at 6 h time point (mean ± SEM, n = 3; * *p* <0.05 as analyzed with one-way ANOVA). Panel (**C**) includes representative immunofluorescence images showing increased red punctuate fluorescence in cells treated with ox-Alb compared to control. Scale bar = 100 μm. (**D**) Oxidative status assessed by measuring protein carbonylation, -SOH formation, and P38 activation in cells treated with 20 mg/mL of control or ox-Alb. β-Actin was used as a loading control.

To explore the mechanism behind the cytotoxicity of ox-Alb, we examined whether there was a difference in Alb internalization by tubular epithelial cells. For this purpose, we determined the amount of Alb in cell lysates. Figure 4A,B show that ox-Alb was internalized by cells more readily than native Alb, as indicated by higher Alb levels in cell lysates (Figure 4A,B). Consistently, immunofluorescence staining of Alb revealed the presence of more intense punctate fluorescent staining in ox-Alb-treated cells as compared to normal Alb (Figure 4C). These observations thus indicate that ox-Alb was more easily internalized by renal tubular cells than native Alb.

Given that oxidative stress is a common mechanism underlying cell injuries initiated by various factors, we analyzed the possible involvement of oxidative stress in the toxic effect of ox-Alb. As shown in Figure 4D, the enhanced uptake of ox-Alb was associated with increased cellular oxidative stress, evidenced by elevated protein carbonylation, -SOH formation, and the activation of redox-sensitive p38 mitogen-activated protein kinase (MAPK) (Figure 4D), suggesting that ox-Alb induces more severe oxidative stress in renal tubular epithelial cells than native Alb.

### 2.5. Ox-Alb Triggers Ferroptotic Cell Death

**Figure 5:**
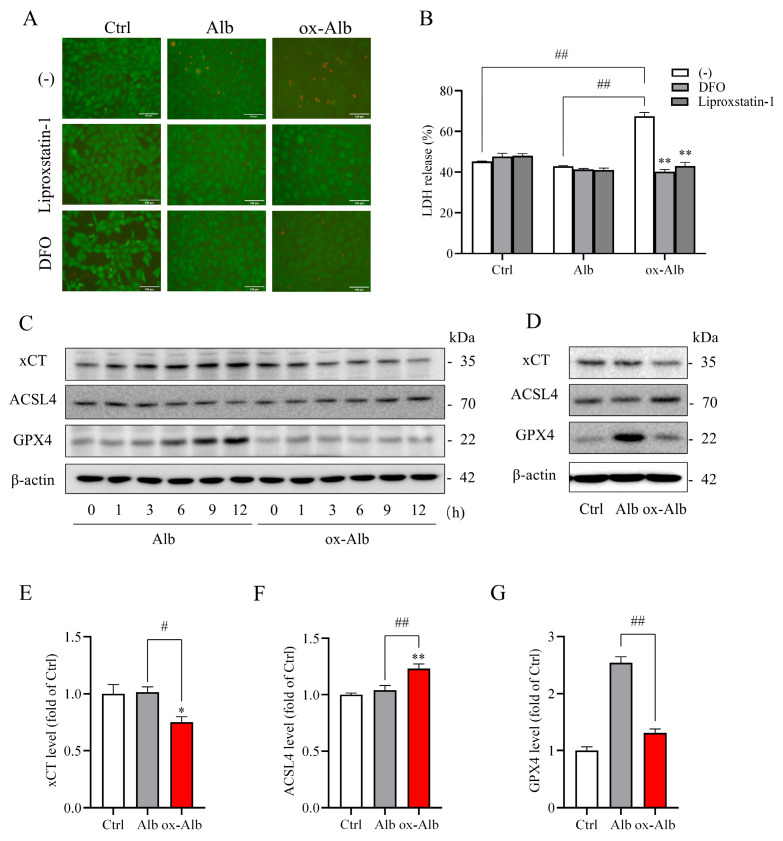
Ferroptotic cell death induced by ox-Alb. (**A**,**B**) Inhibition of ox-Alb-induced cell death by ferroptosis inhibitors. NRK cells were treated with 50 mg/mL control or ox-Alb in the presence or absence of 100 μM DFO or 1 μM Liproxstatin-1 for 24 h. Cell viability was evaluated using Calcein-AM/PI staining (**A**) and LDH release (**B**). Panel B data are the percentage release of LDH against total 100% release expressed as mean ± SEM (n = 4; ** *p* < 0.01 vs. ox-Alb; ## *p* < 0.01, as analyzed with mixed effect analysis). (**C**,**D**) Analysis of ferroptosis-related markers in cells exposed to 50 mg/mL Alb or ox-Alb for specified times (**C**) or 12 h (**D**). Western blot analysis was performed, and protein loading was verified by β-Actin reprobing. Densitometric analysis of bands in (**D**) is shown in (**E**–**G**). Data shown are fold change relative to control and expressed as mean ± SEM (n = 3; * *p* < 0.05 vs. Ctrl; ** *p* < 0.01 vs. Ctrl; # *p* < 0.05; ## *p* < 0.01, as analyzed with one-way ANOVA). Scale bar in (**A**) = 100 mm.

Given the close connection between oxidative stress and ferroptosis, an iron-dependent form of cell death [15,16], we investigated whether ox-Alb could trigger ferroptosis in renal cells. To address this, we evaluated cell death in the presence or absence of ferroptosis inhibitors in cell cultures and examined ferroptosis markers in cellular lysates. As shown in Figure 5A,B, ferroptosis inhibitors, deferoxamine (DFO, an iron chelator), and liproxstatin-1 (a lipid peroxidation inhibitor) significantly mitigated ox-Alb-induced cell death, as demonstrated by Calcein-AM/PI staining and LDH release assays.

Consistently, treatment with ox-Alb resulted in a time-dependent modulation of ferroptosis markers, including decreases in cystine/glutamate antiporter (xCT, a cystine transporter that regulates glutathione synthesis) and glutathione peroxidase 4 (GPX4, a key enzyme that protects cells from lipid peroxidation), as well as an increase in Acyl-CoA synthetase long-chain family member 4 (ACSL4, an enzyme that promotes ferroptosis by remodeling cellular lipid composition) (Figure 5C). These changes strongly indicate the induction of ferroptosis. For quantitative analysis, we further assessed these markers at the 12-h time point. Similar to the time course results, ox-Alb induced significant alterations in ferroptosis markers, confirming the activation of ferroptosis (Figure 5D–G).

**Figure 6:**
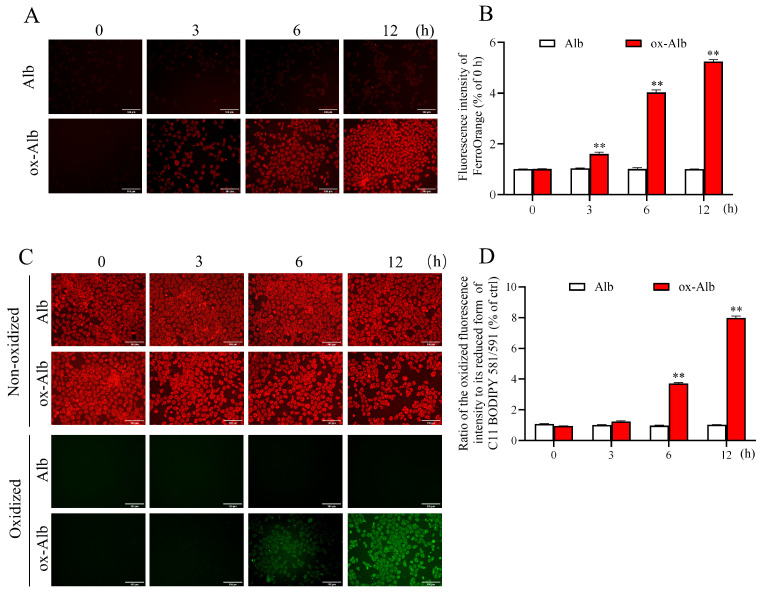
Assessment of intracellular iron levels and lipid peroxides after the treatment with different Alb. Note the increased red fluorescence for iron (**A**) and green fluorescence for oxidized lipid peroxidation (**C**) in cells treated with ox-Alb. Densitometric analysis of the cellular fluorescent signal in (**A**,**C**) is shown in (**B**,**D**). Data are mean ± SEM (n = 4; ** *p* < 0.01 vs. Alb, as analyzed as one-way ANOVA). Scale bar = 100 mm.

To further confirm the occurrence of ferroptosis, we assessed intracellular iron levels and lipid peroxidation, two hallmark features of ferroptosis [15,16]. Using the FerroOrange probe, which selectively binds to intracellular Fe^2+^, we observed a rapid and pronounced increase in intracellular iron levels upon treatment with ox-Alb. This change was visualized as intensified red fluorescence signals, as shown in Figure 6A,B. Simultaneously, lipid peroxidation was detected using the BODIPY 581/591 C11 probe, a fluorescent dye that shifts its emission spectrum from red to green in response to lipid peroxidation. Treatment with ox-Alb led to a significant increase in lipid peroxidation, as evidenced by a shift from the non-oxidized lipid form to the oxidized lipid form (Figure 6C). The ratio of the oxidized fluorescence intensity to its reduced form was significantly increased (Figure 6D). These findings demonstrated the existence of iron accumulation and oxidative lipid damage, further substantiating the induction of ferroptosis by ox-Alb.

Collectively, these observations indicate that ox-Alb, but not normal Alb, induces oxidative stress and causes a ferroptotic cell death in cultured renal tubular cells.

### 2.6. Ox-Alb Exacerbates DOX-Induced Renal Injury In Vivo

**Figure 7:**
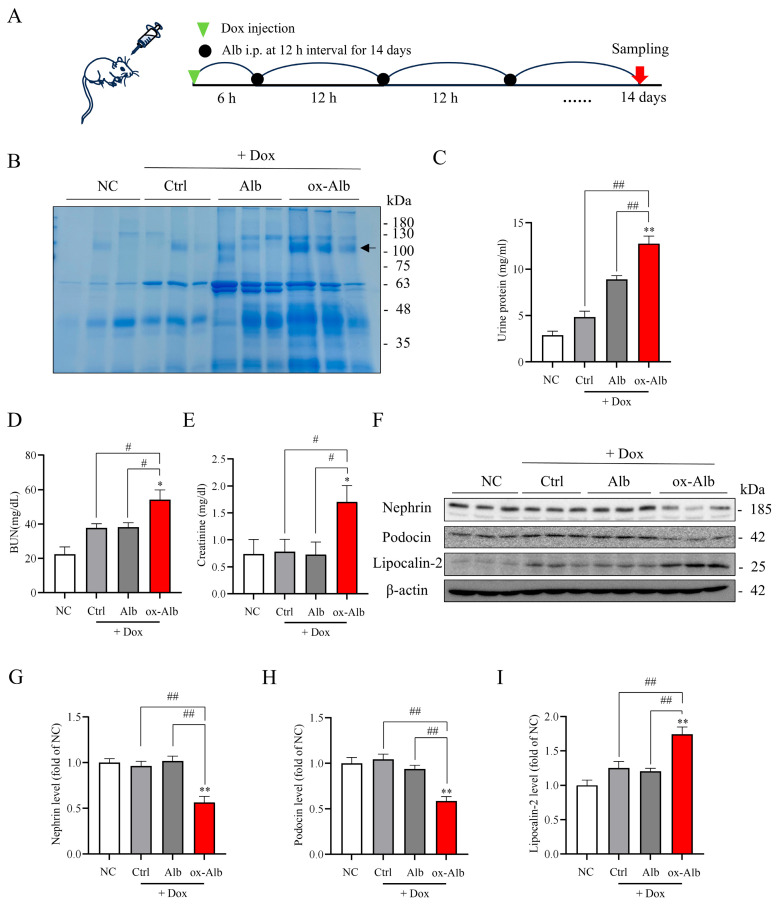
Effects of ox-Alb on DOX-induced renal injury. (**A**) Experimental outline. Mice received an intraperitoneal injection of 20 mg/mL DOX followed by Alb or ox-Alb [2 mg/g body weight (BW)] every 12 h for 14 days. Renal function and redox status were analyzed in blood and kidney samples. (**B**–**E**) Impact of Alb administration on renal function, with measurements of urinary protein (**B**,**C**), BUN (**D**), and creatinine (**E**) on day 14; data shown in (**C**–**E**) are mean ± SEM [n = 6; * *p* < 0.05 vs. NC; ** *p* < 0.01 vs. normal control (NC); # *p* < 0.05; ## *p* < 0.01, as analyzed with one-way ANOVA]. Note the appearance of high MW urinary proteins in mice receiving ox-Alb, as shown in (**B**) (arrow). (**F**–**I**) Analysis of nephrin, podocin, and lipocalin-2 levels, with representative blot images in (**F**) and densitometric analysis in (**G**–**I**). Data shown are mean ± SEM and expressed as fold change against NC (n = 6; * *p* < 0.05 vs. NC; ** *p* < 0.01 vs. NC; # *p* < 0.05; ## *p* < 0.01, as analyzed with one-way ANOVA).

To determine the role and mechanism of ox-Alb in the progress of renal disease, we examined the effects of ox-Alb on DOX-induced kidney injury [22]. The purpose of using DOX was to induce glomerular injury and proteinuria so that ox-Alb could be filtered via the glomerular barrier and accumulate in the renal tubules, exerting its pathological effects. Figure 7A shows the experimental outline. Mice were injected with 20 mg/kg DOX followed by an injection of 2 mg/g control normal and ox-Alb for 14 days. Urine, serum, and kidneys were collected to determine the changes in renal function, structural proteins, and ferroptotic markers.

In our preliminary experiments, we observed that DOX induced an elevation in urinary protein on day 3. This effect of DOX was transient and disappeared after 6 days, indicating that the toxicity of DOX on the kidney was transient and could be self-recovered. As shown in Figure 7B–E, DOX only slightly increased urinary protein, BUN, and creatinine on day 14. However, co-administration with ox-Alb significantly worsened renal dysfunction, as evidenced by the appearance of high molecular weight (MW) proteins (Figure 7B) in urine, associated with the elevated levels of urinary protein (Figure 7C) and significantly increased BUN and creatinine (Figure 7D,E). The occurrence of high MW proteins in the urine indicated damage to the glomerular filtration barrier. In consistence with this notion, the protein level of nephrin and podocin, two key components of the glomerular slit diaphragm in podocytes, were significantly reduced in the ox-Alb group (Figure 7F–I). In addition, lipocalin-2, a well-used renal tubular injury marker [23,24], was also significantly elevated. These results indicate the existence of both glomerular and tubular cell injury.

**Figure 8:**
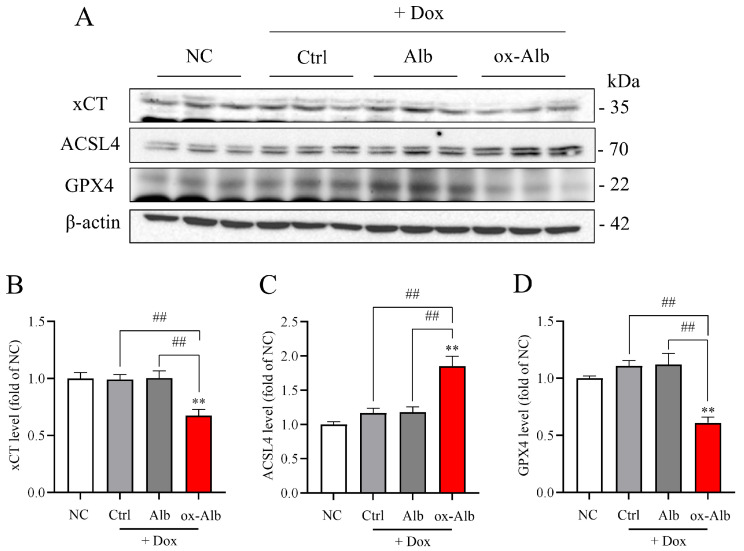
Induction of renal ferroptotic changes by ox-Alb. Mice were treated the same as in Figure 7. Kidney lysates were analyzed for ferroptotic markers xCT, ACSL4, and GPX4. Representative blots are shown in (**A**) with quantification in (**B**–**D**). Data represent fold changes relative to control, mean ± SEM (n = 6; ** *p* < 0.01 vs. NC; ## *p* < 0.01, as analyzed with one-way ANOVA).

Moreover, in accordance with the induction of ferroptosis in vitro, ox-Alb also induced renal ferroptosis in vivo, as evidenced by the decreased levels of GPX4 and xCT, as well as the increased level of ACSL4 (Figure 8A–D).

### 2.7. Ox-Alb Induces a Local and Systemic Oxidative Stress

**Figure 9:**
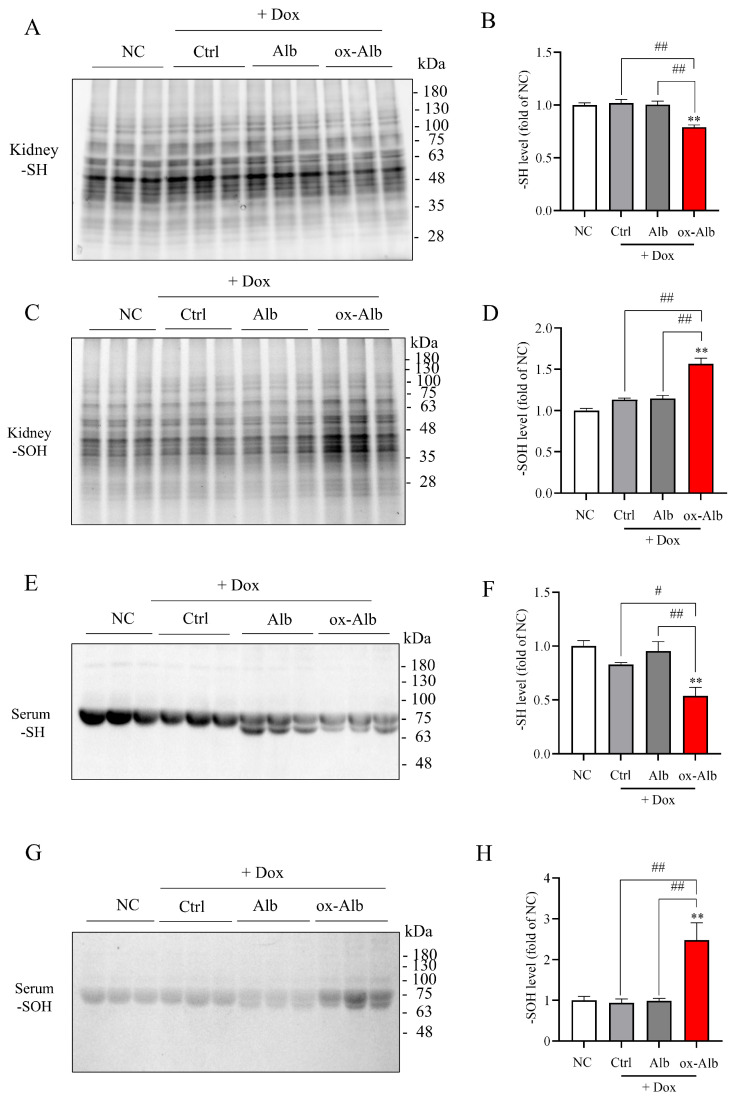
Local and systemic oxidative stress induced by ox-Alb. Following the same DOX and Alb treatment protocol, changes in renal (**A**–**D**) and serum (**E**–**H**) protein oxidation were assessed for -SH and -SOH levels. Representative blot images (**A**,**C**,**E**,**G**) and densitometric quantification (**B**,**D**,**F**,**H**) are provided, with data expressed as fold change relative to control (mean ± SEM; n = 6; ** *p* < 0.01 vs. NC; # *p* < 0.01; ## *p* < 0.01, as analyzed with one-way ANOVA).

To explore the potential mechanisms underlying the in vivo effect of ox-Alb, we also determined the changes in renal and serum protein oxidation after ox-Alb administration. Figure 9A–D show that the administration of DOX at the concentration of 20 mg/kg for 14 days did not significantly affect renal and serum protein oxidation. However, in the mice treated with DOX together with ox-Alb, renal and serum protein oxidation were increased considerably, as evidenced by the increased -SOH and reduced -SH in serum proteins and renal lysates (Figure 9E–H). This effect was not observed in mice co-treated with control Alb. These observations indicate that ox-Alb causes renal and systemic oxidative stress.

## 3. Discussion

**Figure 10:**
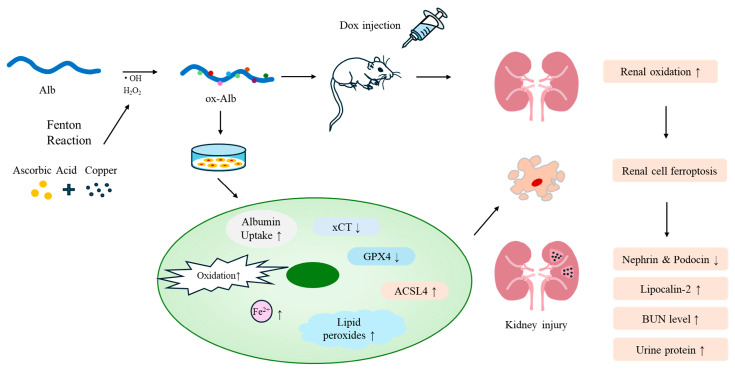
Schematic depiction of the effects and mechanisms of ox-Alb in renal cell injury. ox-Alb is made by exposing Alb to ROS made by AA plus Cu^2+^, which generates H_2_O_2_ and hydroxyl radicals via the Fenton reaction. In cell culture, ox-Alb causes intracellular Alb accumulation, induces oxidative stress, and triggers ferroptotic cell death. In DOX-induced kidney injury, ox-Alb induces oxidative stress, aggerates renal dysfunction, and damages renal cells via a mechanism involving ferroptosis. The small upward and downward arrows denote an increase and a decrease, respectively.

Oxidative stress is a prevalent mechanism underlying various kidney diseases [2,3]. While the damaging effects of ROS on cellular components are well-documented, an expanding body of evidence underscores the pathogenic role of oxidatively modified proteins, particularly oxidized serum Alb, in renal injury. This study offers direct evidence that ox-Alb induces ferroptotic cell death in renal cells and contributes to the progression of kidney disease. The main findings have been schematically depicted in Figure 10. These findings enhance our understanding of the roles and mechanisms through which oxidized proteins contribute to renal injury, positioning ox-Alb as an important mediator that links protein oxidation to renal damage and disease progression. 

Numerous studies have reported increased serum Alb oxidation in various experimental kidney diseases, including diabetic nephropathy, CKD, and drug-induced nephrotoxicity [5,7,8,18]. Consistent with these reports, we observed elevated serum protein oxidation, particularly of Alb, in mouse models of AKI induced by I/R and AA plus Cu^2+^. Elevated levels of circulating ox-Alb have also been documented in patients with renal diseases, correlating positively with the severity of conditions such as diabetic nephropathy and various stages of CKD. ox-Alb has been proposed as a biomarker reflecting in vivo oxidative status and predicting the progression of multiple diseases, including kidney diseases [5,7,8,9,12,18]. While prior studies have primarily focused on correlative associations, our research establishes a causal link between ox-Alb and renal cell injury by directly demonstrating its cytotoxic effects and elucidating the underlying mechanisms.

Oxidative stress, characterized by elevated ROS production (H_2_O_2_, hydroxyl radicals, and superoxide), plays a central role in kidney diseases [2,3]. ROS initiate a cascade of detrimental effects, including inflammation, direct cellular injury, and biomolecule modification [25]. Reciprocally, the inflammation and tissue damage could further exaggerate oxidative stress, creating a vicious cycle that propels disease progression. In the kidney, this cycle is further intensified by the accumulation of uremic toxins, which promote inflammation and oxidation, thus inducing severe serum protein oxidation [26].

In this study, we induced pathological Alb oxidation by exposing Alb to AA plus Cu^2+^. This combination is known to produce large amounts of multiple ROS, including H_2_O_2_, hydroxyl radicals, and superoxide, through metal-catalyzed Fenton chemistry [20,27]. Indeed, AA plus Cu^2+^ resulted in H_2_O_2_ generation and caused increased protein carbonylation, -SOH formation, and decreased free -SH levels, indicative of the induction of protein oxidation. Of note, although carbonyl formation and -SH oxidation have been well-established as markers of protein oxidation, further studies utilizing high-resolution mass spectrometry or chemical analyses are warranted to identify specific side chain modifications and their potential consequence on Alb structure and function [10,11]. In this study, we selected AA plus Cu^2+^ to induce Alb oxidation. The selection is because AA and Cu^2+^ are commonly used nutritional supplements and have been previously reported by our group to induce oxidative renal cell injury in vivo and in vitro [20]. The results obtained from the ox-Alb generated from this approach could also provide novel mechanistic insight into our previous report regarding the renal injury induced by AA plus Cu^2+^.

The exposure of cultured tubular epithelial cells to ox-Alb caused cell death, which was associated with a marked intracellular accumulation of ox-Alb, suggesting the potential disruption of normal protein handling mechanisms. Earlier studies have shown that renal tubular cells possess sophisticated protein uptake and processing machinery, including megalin/cubilin-mediated endocytosis [28,29]. Our findings suggest that oxidative modifications may alter the normal interaction between Alb and these cellular systems, leading to abnormal accumulation and subsequent cellular stress. Consistent with this notion, enhanced endocytosis of modified Alb has been previously reported. For example, covalently modified Alb was preferentially endocytosed in proximal tubular epithelial cells compared to native Alb [30]. Currently, the exact mechanisms underlying the increased accumulation of ox-Alb in cells are unclear; further investigation is needed in the future.

The accumulated proteins in tubular cells resulted in the generation of ROS and oxidative stress, as shown by the increased protein carbonylation and sulfenylation [11], as well as the activation of the redox-sensitive p38 MAPK signaling. The induction of oxidative stress, particularly p38 activation, has been implicated in protein overload-induced cytotoxicity in proximal tubular cells [31]. p38 activation has also been documented to mediate various stress responses and cause both apoptotic and non-apoptotic cell death [32,33]. These events could be critically involved in ox-Alb-induced cell injury.

Our study identified ferroptosis as the primary mechanism underlying ox-Alb-induced cell death. Ferroptosis, a distinct form of regulated cell death, is characterized by iron-dependent lipid peroxidation and the accumulation of ROS within cells. It occurs when antioxidant defenses fail to prevent lipid peroxidation, ultimately causing membrane damage and cell death. This process has emerged as a critical mechanism in the pathogenesis of various diseases, including kidney diseases [15,16,33]. In this investigation, we observed that ox-Alb increased intracellular iron levels and lipid peroxidation. Notably, the cytotoxic effects of ox-Alb were significantly attenuated by treatment with the iron chelator DFO or the lipid peroxidation inhibitor liproxstatin-1, indicating an involvement of ferroptosis.

Further supporting the role of ferroptosis, ox-Alb treatment significantly reduced the levels of GPX4, a key regulator of ferroptosis that prevents lipid peroxidation by converting lipid hydroperoxides into non-toxic lipid alcohols. Additionally, ox-Alb decreased the expression of xCT, impairing glutathione synthesis and weakening cellular antioxidant defenses. Conversely, ox-Alb elevated the levels of ACSL4, a pro-ferroptotic enzyme that facilitates the incorporation of polyunsaturated fatty acids (PUFAs) into phospholipids. This modification makes cellular membranes more prone to peroxidation, thereby amplifying ferroptosis by promoting the formation of oxidizable lipid substrates. These findings provide strong evidence that ox-Alb activates the ferroptotic cell death pathway.

Of note, we have used LDH release as a marker to assess cell death. As LDH is released upon the disruption of the cell membrane, it occurs in various cell types of cell death, including necrosis, apoptosis, and ferroptosis [34]. The almost complete inhibition of LDH release by DFO and liproxstatin-1 suggests that ferroptosis is the primary form of cell death induced by ox-Alb. It is important to note that Alb overload has been reported to induce apoptosis in renal cells [13,32]. In this experimental setting, we did not observe a pronounced cytotoxicity from normal Alb, and the cell death induced by ox-Alb was mainly due to ferroptosis. The question occurs as to how to reconcile the discrepancy. One possibility is that ox-Alb-induced cell death is caused by mechanisms different from normal Alb. Indeed, ox-Alb was more easily endocytosed by tubular cells and exhibited much more potent cytotoxicity to renal cells. It is also possible that ox-Alb also induced apoptosis to an extent much less than ferroptosis. Growing evidence shows that different forms of cell death may occur simultaneously or transit into one another depending on the cellular environment and stimuli [35,36]. Thus, the existence of other types of cell death in our system cannot be wholly excluded. More detailed analyses are needed in the future.

In consistence with the cell culture experiments, our in vivo results show that ox-Alb also exacerbated DOX-induced nephropathy in mice and propagated kidney damage through similar pathways defined in vitro. Specifically, the co-administration of preformed ox-Alb worsened the renal function decline, tubular injury, and ferroptotic changes induced by DOX. Furthermore, the increased proteinuria and decreased protein expression of the podocyte markers nephrin and podocin indicated that ox-Alb may also inflict damage on podocytes in vivo through comparable mechanisms. ox-Alb also induced systemic and renal oxidative stress in vivo. These findings collectively validate the pathogenic effects of circulating ox-Alb via the impairment of antioxidant defenses and the activation of ferroptotic mechanisms.

It is worth noting that we have used different approaches to investigate the in vitro and in vivo pathological effects of ox-Alb. In vitro, ox-Alb was directly added to the culture medium to study its cellular effects. In vivo, however, ox-Alb was administered to mice pretreated with a single injection of DOX, a well-known inducer of kidney injury [22]. This experimental design was based on several considerations. First, our preliminary studies showed that intraperitoneal injections of normal Alb or ox-Alb at the concentrations used did not result in significant changes in urine protein levels or glomerular injury markers for up to 10 days, suggesting that Alb alone, used in our experimental settings, was insufficient to cause overt renal injury. Second, it is widely recognized that the pathological effects of Alb on renal tubular cells require its interaction with tubular cells via reabsorption, a process mediated by specific receptors and endocytosis [28,29,37]. For this to occur, Alb needs to be filtered through the glomerular barrier. Third, in clinics, the pathological effects of Alb in patients with kidney diseases are often associated with pre-existing glomerular damage and proteinuria. To mimic these pathological conditions, we used DOX to induce transient glomerular injury and proteinuria at an early stage, enabling ox-Alb to be filtered and accumulated in the renal tubules. This treatment allowed us to study its role in promoting tubular cell injury and advancing renal disease. Our findings indicate that the co-administration of DOX could be a practical model for studying the effect of Alb on renal disease progression. This approach offers a more efficient and clinically relevant way to study the pathological role of Alb on the progression of renal diseases.

Our findings offer novel insights into the pathogenic role of ox-Alb in renal cell injury. We demonstrated that ox-Alb induces renal tubular cell damage and promotes the progression of renal diseases through mechanisms involving oxidative stress and ferroptotic cell death. Since oxidative stress is a common underlying mechanism in numerous pathological conditions, and oxidized serum proteins have been detected in various oxidative stress-centered diseases [5,8,11], the effects and mechanisms identified in this study may also be relevant to other pathological contexts. For instance, oxidized circulating and dietary proteins are consistently present in the vascular and intestinal systems, which may contribute to the initiation and development of cardiovascular and intestinal diseases. Supporting this perspective, our preliminary experiments showed that ox-Alb also caused damage to intestinal epithelial Caco-2 cells in culture (Supplementary Figure S1). Further investigation into the effects and mechanisms of oxidized proteins on these cells, as well as on other cell types involved in macromolecule clearance, such as liver sinusoidal endothelial cells (LSECs) [38], could be an intriguing direction for future research.

While our study provides direct experimental evidence indicating an involvement of ox-Alb in kidney injury, several important questions remain. The specific chemical modifications of Alb responsible for its cytotoxicity, the molecular pathways by which it disrupts iron metabolism and lipid peroxidation to drive ferroptosis, and the potential involvement of other cell death modalities require further investigation. Additionally, the pathogenic effects of other oxidized biomolecules, such as lipids or nucleic acids, and the role of circulating ox-Alb in the progression of human kidney disease warrant exploration. It is also worth mentioning that, while our data strongly support albumin as a major target of oxidation in serum during such injury, ox-Alb is indeed one component within a complex pathological milieu. Factors such as cell-free heme, labile iron, direct reactive oxygen species (ROS) effects, and even excessively filtered non-oxidized albumin can also contribute to renal damage. The role of ox-Alb should be placed within this broader context, thereby providing a more comprehensive perspective on the pathogenesis of oxidative kidney injury.

## 4. Materials and Methods

### 4.1. Materials

Bovine serum Alb (BSA, Fraction V) was sourced from Iwai Chemical Company (Tokyo, Japan). Copper (II) sulfate (CuSO_4_; Cu^2+^) was procured from Fujifilm Wako Pure Chemical Corporation (Osaka, Japan). DOX was acquired from Tokyo Chemical Industry Co., Ltd. (Tokyo, Japan). Anti-ACSL4 antibodies obtained from Proteintech Genomics (San Diego, CA, USA), and anti-xCT antibodies were purchased from Novus International Inc. (Oakville, ON, Canada). Anti-GPX4, anti-β-actin, and anti-p-P38 antibodies were supplied by Cell Signaling Technology (Beverly, MA, USA). The BUN kit was from Thermo Fisher Scientific (Rockford, IL, USA). DFO and liproxstatin-1 were obtained from Cayman (Ann Arbor, MI, USA). L-AA, maleimide, and other chemicals were sourced from Sigma-Aldrich (St. Louis, MO, USA).

### 4.2. Cell Culture

Rat renal tubular epithelial NRK-52E and human intestinal epithelial Caco-2 cells (ATCC, Rockville, MD, USA) were cultured in Dulbecco’s Modified Eagle Medium (DMEM)/F12 supplemented with 5% Fetal Bovine Serum (FBS) and 1% antibiotic–antimycotic solution under a humidified atmosphere of 5% CO_2_/95% air at 37 °C. For experimental procedures, cells were seeded into culture plates with a medium containing 0.5% FBS and exposed to various stimuli.

### 4.3. Animal Experiments

Male C57BL6/J mice aged 7–8 weeks were housed under a controlled temperature with a 12 h light–dark cycle with ad libitum access to food and water. All animal procedures were approved by the Animal Care and Use Committee of Yamanashi University (A3-583) and adhered to established guidelines and regulations.

For experiments relating to the effect of ox-Alb on DOX-induced kidney injury, the mice were grouped into four groups: control, DOX, DOX plus Alb, and DOX plus ox-Alb, each consisting of three mice. DOX (20 mg/kg) was administered via intraperitoneal injection. Saline was used as a control. Six hours post-administration, 2 mg/g body weight of Alb or ox-Alb was administered abdominally every 12 h for 14 days. On day 14, the mice were euthanized, and their blood and kidneys were collected and stored at −80 °C for further analysis. Notably, the same procedure for the mouse experiments was repeated independently twice, yielding similar results. The combined data from two of these sets (n = 6 per group) are presented.

For studies with AKI induced by renal I/R, mice models were established, as we recently reported [19]. Briefly, the renal pedicle (artery, vein, and ureter) of both kidneys were clamped for 40 min with clips, followed by removing the clips to recover the normal blood flow. After 24 h, mice were anesthetized, and serum was collected to analyze renal function and the status of oxidation.

For the induction of AKI with AA plus Cu^2+^, mice were treated as we previously reported [20]. Briefly, AA (100 mg/kg) and Cu^2+^ (1 mg/kg) were administrated at the volume of 200 mL via oral gavage twice per day for 6 days. Mouse serum was collected and assayed for serum status of oxidation. For control, the same volume of saline was used as the untreated control.

### 4.4. Western Blot Analysis

Cellular proteins were extracted using a 1x SDS lysis buffer (62.5 mM Tris-HCl, 2% SDS, 10% glycerol), while tissue samples were homogenized in an RIPA buffer supplemented with freshly added protease inhibitor cocktail. Protein concentrations were determined using the Micro BCA Protein Assay Kit (Thermo Fisher Scientific, Waltham, MA, USA). Proteins were resolved on a 10% SDS-PAGE gel, transferred to PVDF membranes, and blocked with either 5% skim milk or 3% BSA in 0.1% Tween-20 PBS. Membranes were incubated overnight with primary antibodies (nephrin, Cat. #sc-28192; podocin, Cat. #sc-21009; Santa Cruz Biotechnology; xCT, Cat. #422688; ACSL4, Cat. #22401-1-AP; GPX4, Cat. #52455) at appropriate dilutions, washed, and exposed to peroxidase-conjugated secondary antibodies. Protein bands were detected using enhanced chemiluminescence (Nacalai Tesque; Kyoto, Japan) and imaged with a Fujifilm LAS-1000 analyzer (Fuji Film, Tokyo, Japan). Band intensities were quantified using NIH ImageJ software, accessible at https://imagej.net/ij/ (accessed on 20 November 2024). Protein loading consistency was confirmed via EZ blue staining or β-actin probing.

### 4.5. Preparation of ox-Alb

BSA, dissolved in distilled water at 400 mg/mL, was used for oxidative modification. The prepared BSA solution was incubated with 0.4 M AA and 2 mM Cu^2+^ at 4 °C for 48 h, followed by dialysis against distilled water or PBS in a dialysis cassette (7000 MWCO, 3–12 mL; Thermo Fisher Scientific, Waltham, MA, USA) with at least 7 changes of dialysis solution to remove the unreactive chemicals completely. The thoroughly dialyzed BSA was collected and assayed for protein concentration, examined for the changes in the amount of -SH groups, and stored at −80 °C until use.

### 4.6. Determination of Free -SH Groups with Maleimide-Labeling Assay

The -SH levels in serum and kidneys were determined with a maleimide-labeling assay with the reported protocol [19,20]. Briefly, proteins from serum and renal tissues were exposed to 5 μM Alexa Fluor 680 C2 maleimide at 4 °C for 2 h. Subsequently, the labeled proteins were separated by SDS-PAGE, and the fluorescent signal in the band was captured using a Fujifilm LAS-4000 analyzer (Fuji Film, Tokyo, Japan).

### 4.7. Determination of -SOH

The formation of -SOH in proteins was determined as previously described [19,39]. Serum samples were diluted at 100 folds, while cell and tissue lysates were prepared at 5 mg/mL. Proteins at 10–50 μg were incubated with 1 mM dimedone for 20 min at RT. After mixing with a five-fold non-reducing sample buffer, the samples were separated by SDS-PAGE and subjected to immunoblotting analysis using an anti-cysteine sulfenic acid antibody.

### 4.8. Serum Alb Depletion Experiment

Serum collected from I/R mice was treated with a commercially available Alb depletion reagent to remove serum Alb (Cat. No. WA-013, MINUTE Albumin Depletion Reagent for Plasma and Serum, Invent Biotechnologies, Inc., Plymouth, MN, USA). The depletion was performed following the manufacturer’s instructions. Briefly, 50 μL of mouse serum was thoroughly mixed with an equal volume of the Alb depletion reagent. The mixture was centrifuged at 13,200 rpm for 5 min, and the supernatant containing Alb was discarded. The remaining pellet was resuspended and used for the determination of the efficacy of Alb elimination via EZ blue staining, and its influence on -SH signals was determined with a maleimide-labeling assay.

### 4.9. LDH Release Assay

LDH release was quantified using a commercial kit according to the manufacturer’s guidelines (LDH Cytotoxicity Detection Kit, TaKaRa Biomedicals, Otsu, Japan). Briefly, cells cultured to 80–90% confluence in a 96-well culture plate were exposed to various stimuli for the indicated times. After using centrifugation to pellet the detached cells, the supernatant was carefully collected and analyzed for LDH activity. The supernatant was mixed with an equal volume of assay buffer and incubated at room temperature (RT) for 30 min to allow the reaction to proceed. The absorbance of the resultant red color was measured at 490 nm using a spectrophotometer (Spectra Max 340, Molecular Devices Ltd., Sunnyvale, CA, USA). LDH release was quantified as a percentage of the total release made by treating cells with 2% Triton X-100 for 10 min.

### 4.10. Calcein-AM/PI Staining

Live/dead staining was performed using a Calcein-AM/PI kit (Dojindo, Kumamoto, Japan) according to the manufacturer’s instructions. Briefly, cells in a 96-well plate were treated under experimental conditions. Following treatment, the cells were exposed to a staining solution containing 2 mM Calcein-AM and 1 ug/mL PI solution for 10–20 min. Subsequently, the green fluorescent living cells from Calcein-AM and the red fluorescent dead cells from PI were visualized and captured under a fluorescence microscope (OLYMPUS IX71, Tokyo, Japan).

### 4.11. Determination of H_2_O_2_ Concentration

The concentration of H_2_O_2_ was measured using a kit from Cayman Chemical Company (Ann Arbor, MI, USA, 600050) following the manufacturer’s instructions. In brief, samples (including PBS control, AA, and Cu^2+^ at specified concentrations) were added into a 96-well plate and allowed to react with assay buffer for 5 min, followed by the addition of enzyme solution. After 30 min, the resulting fluorescence was measured using a SpectraMax^®^ GEMINI EM (Sunnyvale, CA, USA) at an excitation wavelength of 530 nm and an emission wavelength of 590 nm.

### 4.12. Determination of Intracellular Iron Using FerroOrange

Intracellular iron levels were assessed using FerroOrange (Dojindo, Kumamoto, Japan), a fluorescent probe that specifically detects labile Fe^2+^ within cells. Cells were seeded in a 96-well plate and treated under experimental conditions. After treatment, the cells were incubated with 1 µM FerroOrange solution at 37 °C for 30 min in the dark. Following incubation, cellular fluorescence was visualized and captured using a fluorescence microscope.

### 4.13. Assay of Lipid Peroxidation with BDP 581/591 C11

Lipid peroxidation was measured using the BDP 581/591 C11 fluorescent probe (Dojindo, Kumamoto, Japan). This probe is reactive to lipid radicals generated during the process of lipid peroxidation, thus undergoing a change in its fluorescence properties, shifting from red emission (590 nm) to green emission (510 nm). Briefly, BDP 581/591 C11 working solution was added into the cells stimulated with various stimuli and incubated at 37 °C for 30 min. After washing the cells, the cellular fluorescent signal was captured with a fluorescent microscope (OLYMPUS IX71, Tokyo, Japan).

### 4.14. Statistical Analysis

Values are expressed as mean ± SEM. A comparison of two groups was made by Student’s *t*-test. For multiple comparisons with the same control (n = 6), one-way ANOVA analyses and mixed-effects analyses were performed. Both analyses were done using Microsoft Excel (Microsoft, Redmond, WA, USA) or GraphPad Prism9 Software (Version 9, GraphPad, San Diego, CA, USA). *p* < 0.05 was considered statistically significant.

### 4.15. Flow Chart Summarizing the Experimental Design

**Figure 11:**
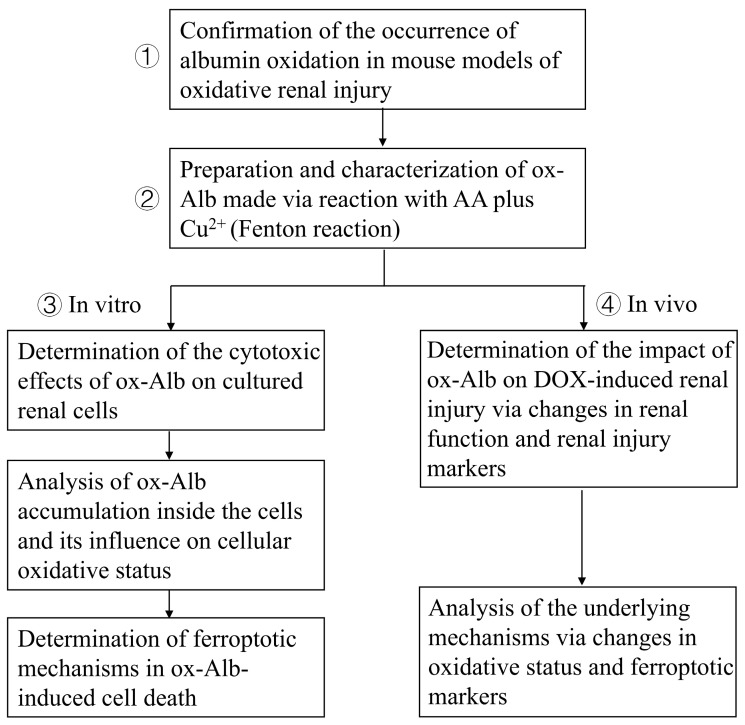
The flowchart of the experimental design and content.

The study aimed to clarify the pathological role and mechanism of ox-Alb in renal tubular cells and renal disease progression. The study includes the following: (1) The determination of Alb oxidation in mouse models: The existence of serum protein oxidation was confirmed in mouse models of oxidative renal injury induced by AA plus Cu^2+^ and I/R. (2) The preparation and characterization of ox-Alb: ox-Alb was prepared by incubating Alb with AA and copper, which initiated a Fenton reaction to generate free radicals, and characterized for its oxidative modifications. (3) The cytotoxic action and mechanism of ox-Alb in cultured renal tubular cells: The cytotoxic effect of ox-Alb on cultured renal tubular epithelial cells was assessed with Calcein-AM/PI staining and LDH release assays, and mechanistic analyses were conducted to assess oxidative stress markers (protein carbonylation and p38 activation), ferroptosis markers (GPX4, xCT, and ACSL4), and the effects of ferroptosis inhibitors (DFO and Liproxstatin-1). (4) In vivo effects of ox-Alb: ox-Alb was administered to DOX-pretreated mice to investigate its effects on the deterioration of renal function (urinary protein levels, BUN, creatinine), structural damage (glomerular and tubular injury markers), and ferroptosis-related molecular changes. A flow chart summarizing the experimental design is provided below (Figure 11).

## 5. Conclusions

In conclusion, our study identifies ox-Alb as a pathogenic factor contributing to kidney injury and highlights ferroptosis as a key underlying mechanism. These findings provide novel insights into the role of ox-Alb in the pathogenesis of oxidative stress-driven kidney diseases. Furthermore, our findings suggest that targeting oxidative stress and ferroptosis may represent a promising strategy for preventing and treating these conditions.

## Data Availability

The data is obtained within the article.

## Supplementary Materials

The following supporting information can be downloaded at: https://www.mdpi.com/article/10.3390/ijms26135924/s1.

## Abbreviations

The following abbreviations are used in this manuscript:

AKI	Acute Kidney Injury
CKD	Chronic Kidney Disease
ROS	Reactive Oxygen Species
NADPH	Nicotinamide Adenine Dinucleotide Phosphate
Alb	Albumin
Cys34	Cysteine Thiol at Residue 34
ox-Alb	Oxidized Alb
DOX	Doxorubicin
AA	Ascorbic Acid
Cu^2+^	Copper (II) Sulfate
I/R	Ischemia/Reperfusion
BUN	Blood Urea Nitrogen
-SH	Sulfhydryl
-SOH	Sulfenic Acid
SEM	Standard Error of the Mean
H_2_O_2_	Hydrogen Peroxide
AM	Acetoxymethyl
PI	Propidium Iodide
LDH	Lactate Dehydrogenase
MAPK	Mitogen-Activated Protein Kinase
DFO	Deferoxamine
MW	Molecular Weight
BW	Body Weight
NC	Normal Control
GPX4	Glutathione Peroxidase 4
xCT	Cystine/Glutamate Antiporter
ACSL4	Acyl-CoA Synthetase Long-Chain Family Member 4
PUFAs	Polyunsaturated Fatty Acids
LSECS	Liver Sinusoidal Endothelial Cells
BSA	Bovine serum Alb
DMEM	Dulbecco’s Modified Eagle Medium
FBS	Fetal Bovine Serum
RT	Room Temperature
