# Supplementary material for: Oxidized Albumin Induces Renal Tubular Cell Death and Promotes the Progression of Renal Diseases Through Ferroptosis
Source: Int J Mol Sci. 2025 Jun 20;26(13):5924. doi: 10.3390/ijms26135924 (PMC12249609; doi:10.3390/ijms26135924)
Supplement: Supplementary file 1 [file ijms-26-05924-s001.zip › ijms-3629989-supplementary.pdf]

# Supplementary Figure 1

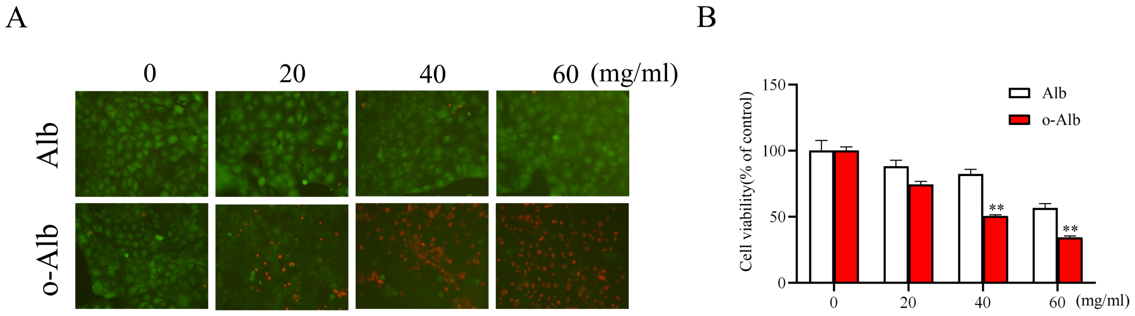


**Supplementary figure S 1.** The cytotoxic effects of oxidized albumin on cultured human intestinal epithelial Caco-2 cells. Caco-2 cells were exposed to the indicated concentrations of normal (Alb) and oxidized albumin (o-Alb) for 24 hours. Cell viability was assessed via Calcein-AM/PI staining (A) and WST assay (B). Data in B are percentage changes relative to respective control (mean ± SE; n=4; ***p*<0.01 vs. Alb).
